# Engineering the Bacterial Laccase CotA for Functional Expression and Dye Decolorization Through Site-Directed Mutagenesis

**DOI:** 10.3390/biology14101335

**Published:** 2025-09-28

**Authors:** Zhiguo Zhou, Shuyuan Yao, Sitie Ying, Mengyan Yu, Zhihua Song, Yongtao Sun, Lisheng Qian, Yue Zhang

**Affiliations:** 1 College of Food Science and Engineering, Anhui Science and Technology University, Chuzhou 233100, China; 2 College of Management, Anhui Science and Technology University, Chuzhou 233100, China; 3 College of Life and Health Sciences, Anhui Science and Technology University, Chuzhou 233100, China

**Keywords:** bacterial laccase, site-directed mutagenesis, copper concentration, expression, enzymatic characteristics, dye decolorization

## Abstract

**Simple Summary:**

In this study, our objective was to enhance a bacterial enzyme known as CotA, which is capable of degrading harmful dyes present in industrial wastewater. Through targeted modifications to the enzyme’s structure, we engineered several variants and evaluated their production efficiency in bacteria, their activity levels, and their efficacy in removing dye pollutants. We discovered that certain modified enzymes exhibited increased activity and superior dye-removal capabilities—specifically, one mutant variant was able to eliminate over 58% of a particular dye within one hour. These findings indicate that judiciously engineered changes to the enzyme can improve its performance, providing a promising biological solution for mitigating water pollution and aiding environmental remediation efforts.

**Abstract:**

The relationship between the structure and function of bacterial laccases has garnered significant research attention thanks to their straightforward molecular structure. Nevertheless, studies examining the impact of an altered molecular structure on the heterologous expression of bacterial laccases in *Escherichia coli* remain scarce. Our research focuses on elucidating the impact of incorporating copper ions into the molecular structure of modified CotA on its exogenous expression in *E. coli* as well as its impact on the significance of the amino acid residues surrounding the internal electron channels and water molecule channels of the enzyme molecule. The results show that single-site mutation may affect the expression of CotA by affecting its soluble expression with different binding capacities for copper ions. In addition, the mutants exhibited different laccase activity levels. The catalytic efficiency of T466A was found to be significantly enhanced, reaching 2.29 times that of the wild type. We used structural models to illustrate the correlation between molecular structure and function after the replacement of three mutation sites with alanine. The reduction of hydrogen bonds may be an important factor influencing Cu^2+^’s binding ability and the water molecule production rate. The T466A mutant exhibited strong decolorization ability for Reactive Blue 19 and Eriochrome Black T with 42.2% and 58.2% decolorization rates after one hour of reaction, respectively. This study demonstrates that the molecular mutation studied influences the CotA expression level, enzyme activity, and dye decolorization.

## 1. Introduction

The structure–function relationships underlying the properties of the bacterial laccase CotA from *Bacillus subtilis* are well documented, rendering the enzyme suitable for protein engineering applications [1,2]. CotA has garnered significant scientific attention because its structural characteristics are simpler than those of its fungal counterparts [3,4]. A distinguishing feature of bacterial laccases is their lack of complex post-translational modifications, such as glycosylation [5], and their susceptibility to site-specific mutagenesis, which is a process that facilitates probing the structure–function relationship of laccases. A substantial body of current research has demonstrated the efficacy of molecular modification techniques in enhancing the properties of CotA, including enzyme activity and dye decolorization [6,7]. However, most studies have focused on the active site or the amino acid residues near T1Cu, and only a few have investigated the amino acids surrounding the electron channel and water molecule channel within the enzyme molecule. This may affect the catalytic efficiency of laccase and the dye decolorization efficiency. Dyes are a very persistent type of pollutant in the environment. Reactive Blue 19 (RB 19), Eriochrome Black T (EBT), and Malachite Green (MG) are anthraquinone, azo, and triphenylmethane dyes, respectively. In particular, RB 19 has been identified as a “model pollutant” in the fields of water treatment, environmental science, and dye degradation research [8,9,10].

A comparison of the gene regulatory expression of fungi and the heterologous expression of bacterial laccases in *E. coli* reveals that the former are more consistently influenced by the bacterial strain, the expression plasmid, and the inducer IPTG [11,12]. This observation establishes a basis for exploring the influence of copper ions on the expression and affinity of enzyme molecules. However, to date, relatively few studies have been conducted to investigate the effect of residue site alteration on bacterial laccase expression, including the amount of copper ions added, soluble expression, or inclusion body formation [13,14]. Furthermore, the catalytic reaction of laccases is facilitated by a four-copper network cluster designated as type 1 (T1), type 2 (T2), or type 3 (T3). These clusters are the sites where catalytic reactions occur, including substrate oxidation, electron transfer, and redox reactions [15,16,17]. The substitution of amino acid residues, particularly those in proximity to copper ion ligands, has been demonstrated to result in variations in expression levels, which can be attributed to the affinity or capture ability of enzyme molecules and copper ions [18,19]. Structural variations may also affect the optimal catalytic conditions and stability of CotA. The substitution of amino acids has been demonstrated to enhance the thermal stability of laccases by introducing salt bridges, hydrogen bonds, hydrophobic interactions, and disulfide bonds. This substitution has been shown to reduce flexibility and eliminate lone pairs of electrons. Amino acid substitution has also been demonstrated to modify the pH adaptability of laccases. For instance, employing specific amino acids to substitute the p*K*_a_ values of the enzyme’s active site and surface-ionizable groups has been shown to affect this modification [20,21]. Molecular modification also serves as a crucial method for altering the application of CotA in dye decolorization, representing a highly promising direction in the industrial utilization of CotA. Therefore, modifications to CotA’s structure and amino acid residue substitutions enable a deeper exploration of the pivotal roles of critical amino acid sites, which include their expression levels, catalytic activities, and capabilities in dye decolorization.

In this study, three mutant strains, I421A, T466A, and K464A, were obtained through targeted mutation transformation. We examined the impact of varying copper ion concentrations on the enzymatic characteristics and laccase expression of the recombinant CotA laccase and its mutants, elucidating the role of copper in modulating these properties. Furthermore, an analytical investigation was undertaken to ascertain the molecular structural variations in the mutant bacterial laccase. The analysis primarily focused on the effects on catalytic activity and dye degradation ability of the mutant bacterial laccase with diverse internal structures. This study aims to refine the induction parameters for recombinant and mutant bacterial laccases by modulating copper ion concentrations, drawing on insights from previous research that demonstrated the significant impact of amino acid replacement on laccase activity, gene expression, and industrial application.

## 2. Materials and Methods

### 2.1. Materials

Growth media were purchased from Sangon (Shanghai, China). The PCR system was from Eppendorf (Shanghai, China). Isopropyl-d-1-thiogalactopyranoside (IPTG) is a product of Biofroxx (Guangzhou, China). The PCR reagents were procured from Beyotime (Shanghai, China). 2,2′-Azino-bis-(3-ethylbenzothiazoline-6-sulfonic acid) diammonium salt (ABTS) was obtained from Bomei (Hefei, China). Reactive Blue 19 (RB 19), Eriochrome Black T (EBT), and Malachite Green (MG) were obtained from Rhawn (Shanghai, China). The procurement of all additional chemical substances was facilitated by Sinopharm Chemical Reagent Co., Ltd. (Shanghai, China).

### 2.2. Bacterial Strains, Plasmids, and Growth Conditions

The competent cells of *E. coli* BL21 (DE3) were incubated using an ice bath, and then the recombinant plasmid pET28a-*cotA* and the recombinant plasmid with other mutant genes were subsequently introduced and incubated with agitation. The transformed expression strain in the suspension was coated and incubated at 37 °C for a period of 12 h. Single colonies were then selected and transferred to LB liquid medium with kanamycin sulfate (with a final concentration of 100 µg/mL), where they were incubated at 37 °C at 180 rpm/min for 12 h. The recombinant plasmid pET28a-*cotA* and its mutant variants in the expression strain were isolated using a plasmid mini-extraction kit and subjected to verification and sequencing. To verify the amplification products, agarose gel electrophoresis was used, and the correct mutated gene sequence was verified through the sequencing process.

### 2.3. Bioinformatics Analysis

The FASTA protein sequence of the CotA laccase (accession: UVZ47122) was obtained from the NCBI (National Center for Biotechnology Information) database (https://www.ncbi.nlm.nih.gov/). The sequence served as the query for all subsequent bioinformatics analyses. Multiple sequence alignment was performed with MAGE. The figure was created using Jalview 2.11.5.0 and T-Coffee (https://www.ebi.ac.uk/jdispatcher/msa/tcoffee?stype = protein (accessed on 9 May 2025)). The molecular representation of the enzyme structures was determined by the PyMOL 2.6.0 viewer. The 3D structure of the laccase (PDB: 1GSK) was retrieved from PDB (https://www.pdbus.org), and the original ligands and water molecules were removed from the laccase protein using PyMOL.

### 2.4. Site-Directed Mutagenesis

The residue Ile421 is close to the electron transfer pathway. It is hypothesized that replacing the amino acid at this position could affect the electron transfer rate and catalytic efficiency. Lys464 and Thr466 are located near the water molecule channel. It is hypothesized that replacing the amino acid at these positions could affect the generation rate of water molecules, thereby influencing the catalytic efficiency of the laccase.

Four sets of primers (Table S1) were designed using the plasmid pET28a (+) containing the wild-type laccase gene as a template.

Screening all the mutants was conducted directly, and the results were subsequently confirmed by DNA sequencing.

### 2.5. Expression and Purification

The recombinant CotA laccase, mutant laccases I421A, T466A, and K464A, and C492A cryopreserved strains were inoculated into LB liquid medium with kanamycin sulfate. The inoculated samples were then incubated with shaking. Thereafter, the activated bacterial solution was transferred to LB liquid medium, which also contained kanamycin sulfate. Culturing was halted once the bacterial concentration reached 0.6 at OD_600_. Subsequently, a solution of 10 µM IPTG and CuSO_4_ was incorporated, which was followed by a period of agitating the culture. Following the incubation period, copper ions were introduced into the medium at concentrations of 0.25 mM, 0.5 mM, 1.0 mM, 1.5 mM, and 2.0 mM, respectively. The amount of added Cu^2+^ was tested on WT-CotA and the mutant under the same conditions. Cell disruption was achieved through sonication on ice, and the subsequent removal of cell debris was facilitated by centrifugation at 14,000× *g* for 18 min at 4 °C. The protein purification process was executed using a His Trap FF crude 5 mL preloaded column, which was integrated with an AKTA purifier chromatography system. Desalting was carried out using Amicon Ultra ultrafiltration tubes (50,000 MWco), with the imidazole solution being substituted by 1× PBS buffer.

### 2.6. SDS-PAGE and Western Blot

The purification effect and protein content were confirmed via SDS-PAGE protein gel electrophoresis. The concentration of the separating gel was set to 12%, the concentration of the concentrating gel was 5%, and the staining procedure used a Brilliant Blue R-250 staining solution. SDS-PAGE protein gels were cut according to the display of rainbow 130 broad-spectrum markers. The primary antibody utilized was an anti-His tag monoclonal antibody, while the secondary antibody was horseradish peroxidase-labeled goat anti-mouse IgG. The Western blot experiment was conducted using 0.1 g and 100 milliliters of *E. coli*, respectively.

### 2.7. Effect of Temperature and pH on Laccase Activity and Stability

Temperatures ranging from 30 to 90 °C were chosen as the optimal temperatures, which were determined by the use of ABTS as a substrate. The samples were subjected to an incubation temperature of 60 °C for a duration of 120 min. The impact of temperature on the residual activity of wild-type CotA (WT-CotA) and its mutants was subsequently evaluated. The influence of pH on laccase activity and stability was ascertained at the optimal temperature using a 10 mM phosphate buffer with a pH range of 2.0 to 10.0. Following a two-hour incubation period, the relative activity of laccase was measured [22]. Experimental measurements were conducted three times.

### 2.8. Decolorization of Dye Assay

The reaction mixture was prepared in 20 mM sodium acetate buffer (pH 3.5, 45 °C) with a final concentration of 20 mg/L for each dye and 0.5 U of each enzyme. The degree of absorption was subsequently quantified at the wavelength at which the dye exhibited its maximal absorption using an enzyme-linked immunosorbent assay (ELISA) reader. This measurement was taken one hour after initiating the reaction. Experimental measurements were conducted three times.

## 3. Results

### 3.1. Mutation Design and Cloning

**Figure 1:**
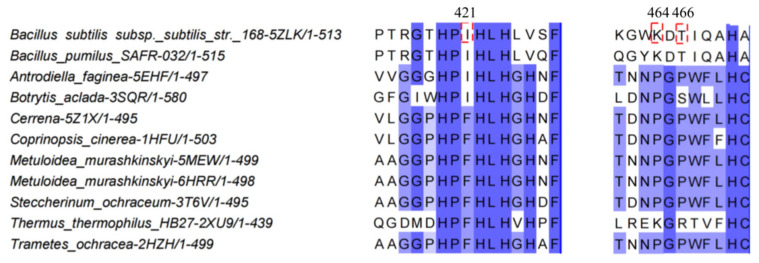
Homology of amino acid sequence around the copper binding sites of multi-copper oxidase.

The CotA laccase from *Bacillus subtilis* 168 is frequently used as a template for sequence comparison with a variety of laccases, including those derived from bacteria and certain fungal species. A 3D model of the laccase was constructed, using Swiss-Model and CotA as the template. The amino acid residues Ile, Lys, Thr, and Cys at 421, 464, 466, and 492 are located in domain 3. In *Bacillus* sp. 168, this domain includes the conserved sequence of Asp465 and His422 to His424, which is the axial ligand of the T2 and T3 copper ions. As illustrated in Figure 1, through multiple sequence alignments, Ile421 in CotA was found to be adjacent to the conserved sequences spanning from 419 to 420. These sequences show a high level of similarity among Bacillus pumilus and various bacterial laccases. However, Phe was identified at the corresponding position in the laccases from most fungi. Moreover, it has been established that 464 and 466 are seemingly indispensable, and the conserved sequences within most laccases feature Pro. Nonetheless, Asp465 in bacterial laccases differs from Gly in most fungal laccases. Furthermore, C492 has been demonstrated to be an irreplaceable conserved locus in previous related studies [16]. To investigate the possible role of these four residues in activity and expression levels with the effect of copper ions, four single mutations (I421A, T466A, K464A, and C492A) with an Ala insertion successfully replaced the *cotA* gene from *Bacillus* sp. 168. Ala insertion is often used to attempt to replace amino acid residue sites because of its smallest structure. Substituting alanine for the original amino acid shortens the side chain, reduces steric hindrance, and exerts a greater spatial effect on the substrate [7]. The plasmids carrying the wild-type and mutated genes were then transformed into *E. coli* BL-21 for expression. Subsequently, the WT-CotA and mutated enzymes were purified to a purity level exceeding 95%, employing the His-Tag labeling method.

### 3.2. Expression and Purification

The four single-point mutations were individually constructed into WT-CotA to investigate the effects of the predicted single-point mutations on soluble protein expression in *E. coli*, and the proteins of WT-CotA and its variants were expressed under the same expression conditions. As shown in Figure S1, the WT-CotA and mutant proteins were subjected to SDS-PAGE analysis. Previous studies reported that the molecular weight of laccase is approximately 67 kDa [23]. SDS-PAGE analysis demonstrated an inconspicuous difference in expression levels between single-point mutants and the WT-CotA, as evidenced by the comparable levels of the laccase in the whole-cell lysis solution. Furthermore, laccase activity assays were conducted on the culture media to ascertain the soluble expression levels of the target enzymes. Compared with the activity of 70.35 U/L ± 23.74 U/L prior to the addition of Cu^2+^, the activity of the WT-CotA enzyme increased significantly, reaching 2207 U/L ± 33.25 U/L, which is approximately 3.5 times higher than the activity observed in other mutants, such as 631 U/L ± 22.25 U/L. I421A demonstrates 2.6-fold higher activity compared to 852 U/L ± 32.6 U/L for T466A and 7.5-fold higher activity compared to 295 U/L ± 13.8 U/L for K464A. This finding suggests that while the overall expression level has remained almost unchanged, the solubility of the expressed protein may have undergone a substantial decline. The specific enzyme activities of the WT-CotA and mutants were also measured (Table 1). Cys492 is a conserved amino acid residue that has been confirmed in previous studies. In the ensuing experimental results, no enzyme activity or dye decolorization ability was detected in the mutant. Therefore, Cys492 does not involve changes in enzyme expression levels and enzyme properties.

**Figure 2:**
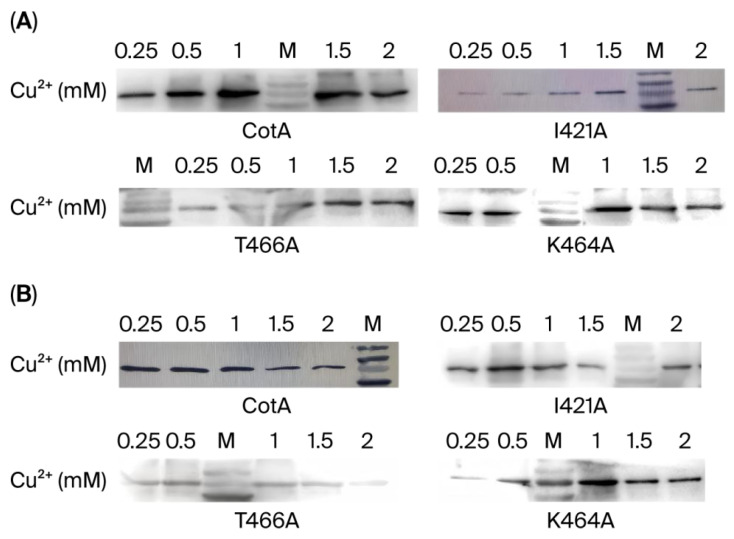
WB of four types of laccase. (**A**) Strain of the same weight. (**B**) Same volume bacterial strain. Original Western Blot figures are included in the Supplementary Materials Figure S2.

To optimize laccase production and soluble protein expression, we examined the effects of varying concentrations of Cu^2+^ on protein expression. BL21 (DE3) cells were cultivated under five distinct concentrations of copper ions (0.25 mM, 0.5 mM, 1.0 mM, 1.5 mM, and 2.0 mM) to assess protein expression. Protein expression analysis was conducted using a Western blot procedure, as illustrated in Figure 2A. The hypothesis posited that with bacteria of an equivalent gram weight (0.1 g), the laccase expression of recombinant CotA, namely the mutant laccase (I421A, T466A, or K464A), reached a maximum level at a copper ion concentration of 1.0 mM or 1.5 mM. Then, predicated on the assumption that the copper ion concentration would be 0.25 mM (Figure 2B), the highest expressions were observed for cell-lysed CotA and K464A with a starter culture of 1 mM CuSO_4_ and I421A and T466A with a starter culture of 0.5 mM CuSO_4_. These culturing conditions were subsequently employed to produce laccases, which were then subjected to a series of functional tests.

### 3.3. Enzymatic Property Analysis

The optimal temperature for the WT-CotA and mutants was determined to be within the range of 30–90 °C. The maximum activity of WT-CotA and I421A was observed following 2 min of incubation with ABTS at a temperature of 80 °C. In contrast, the optimal temperature for T466A was determined to be 70 °C, while K464A exhibited relatively high activity at 90 °C (Figure S3A). The thermostability of these enzymes was studied at a temperature of 60 °C for a period of 240 min (Figure S3B). The mutant demonstrated comparable temperature stability to the WT-CotA. However, after 90 min, the K464A showed an 80% reduction in activity, which was slightly less than the 89% decline seen in the WT-CotA.

The results indicate that the optimal pH of the mutated CotA is not significantly different from that of the WT-CotA (Figure S3C). However, the mutants did not demonstrate pH stability comparable to that of the WT-CotA. In the context of the effects of pH on enzyme activity, it has been observed that under acidic conditions, such as a pH of 3.0, the activity of the WT-CotA enzyme can decrease significantly, with a 60% reduction in activity compared to its activity at pH 5.0. In addition, the mutants’ activity is reduced by 90%. Similarly, mutant enzymes like I421A can experience an even greater decrease in activity, reaching 86% under the same conditions at a pH of 4.0 (Figure S3D). In environments with a pH higher than 7, all the enzymes demonstrated better stability (alkaline conditions).

### 3.4. Dye Decolorization

**Figure 3:**
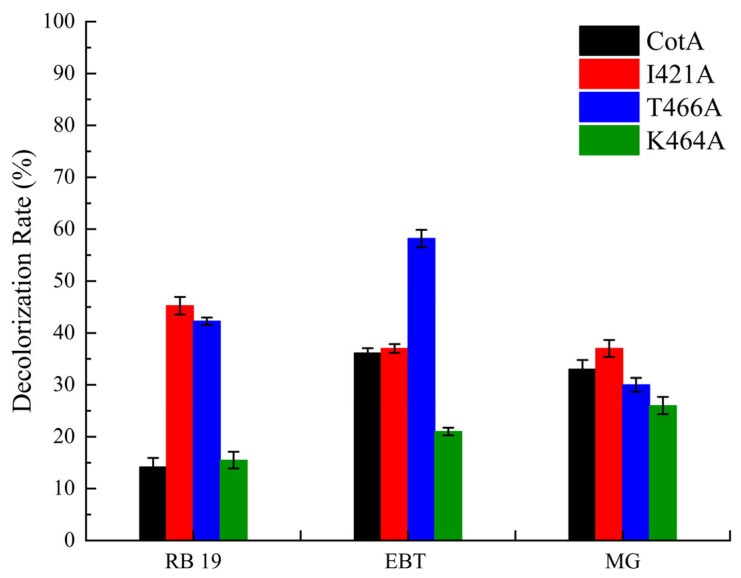
Use WT-CotA and mutant CotA laccase to decolorize RB 19, EBT, and MG. Measure for 1 h under the condition at 37 °C at pH 3.5.

In order to investigate the effect of the enzymes on dye decolorization, three dyes were selected as substrates: RB 19, EBT, and MG. The I421A mutant and T466A exhibited a strong decolorization ability for RB 19 with decolorization rates of 45.24% and 42.26% after one hour of reaction, respectively. In contrast, the decolorization ability of WT-CotA was weaker with a decolorization rate of 14.17% (Figure 3). Furthermore, the decolorization rate of T466A to EBT increased to 58.20%, which is indicative of a superior decolorization rate compared to that of WT-CotA, which had a rate of 36.15%. Furthermore, the WT-CotA and all mutants show a similar decolorization effect for MG at nearly 30%. This might be due to their similar affinities.

## 4. Discussion

**Figure 4:**
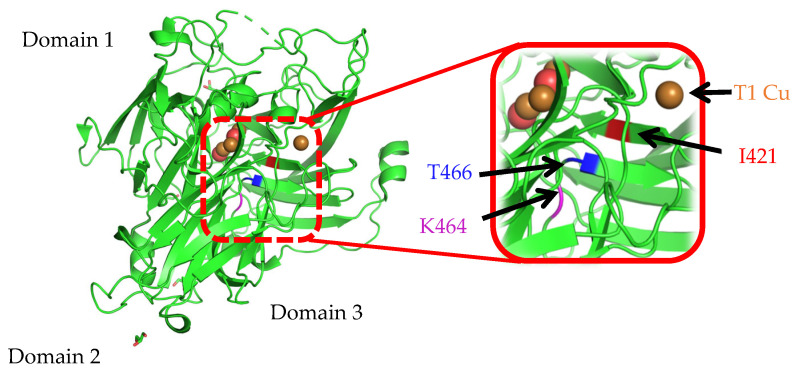
The location of the amino acid mutation site and its distance from the nearest copper ion.

The objective of this study was to explore the potential involvement of three amino acid residues in the C-terminal segment of *Bacillus* sp. 168 laccase CotA, as shown in Figure 4. These amino acid residues are located near the mononuclear copper center, where they play a crucial role in the protein’s structure and function. A comparative analysis of their sequences was conducted, encompassing both horizontal and vertical comparisons. Additionally, the expression levels of CotA and the mutants were investigated through SDS-PAGE and Western blot analysis. Furthermore, the catalytic properties of the mutants were evaluated to assess the impact of these amino acid changes, and the decolorization capacity of the dye was evaluated in comparison.

**Figure 5:**
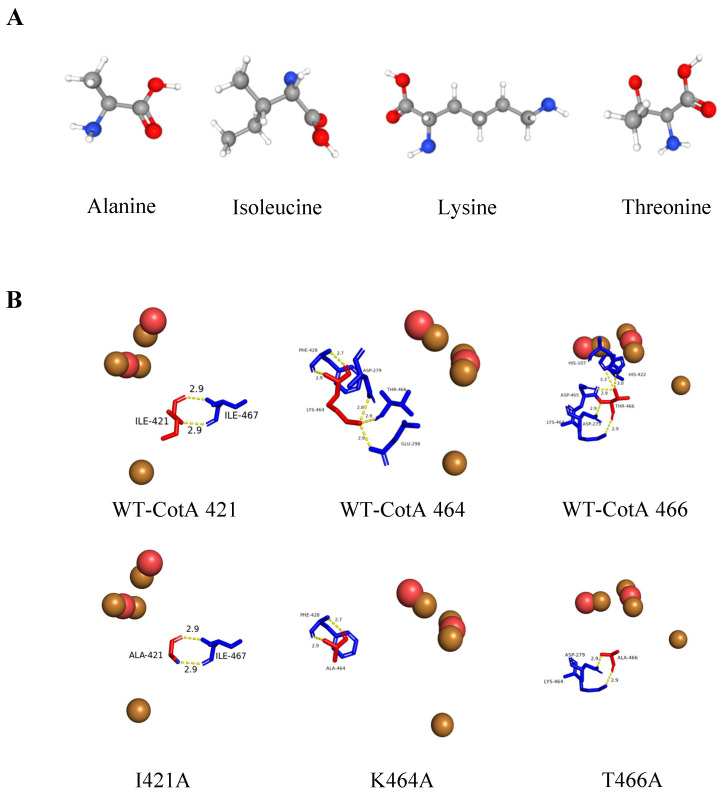
(**A**) A stick model of four amino acids. (**B**) The number of hydrogen bonds in CotA and three mutants, as well as the hydrogen bonds before and after mutation, are indicated by yellow dashed lines. The unit of distance between all amino acid residues is Å.

The region connecting copper ligands is characterized by fixed combinations, including several amino acid residues such as Cys, His, and Met (Leu/Phe). These amino acids, which are present in all multi-copper oxidases, exhibit a mixed pattern with one to three amino acids [24]. In addition, a network of water channels has been observed to facilitate communication between the trinuclear copper center and the external environment. These channels are formed by the synergistic interaction of three distinct domains, thereby establishing a conduit for molecular exchange, and the dioxygen approaches the trinuclear copper center through these channels [25]. It has been posited that I421 in *Bacillus* sp. CotA (equivalent to Phe in most fungal laccases) is situated at the surface of the channel between T1 copper and the trinuclear center. It is further postulated that I421 may form hydrogen bonds with the solvent, which is a process that is deemed to be of significant importance for electron transfer [26]. Previous studies indicated that conserved domains from 419 to 420 and 422 to 424 have a major influence on the catalytic activity of the laccase [16]. In the WT-CotA (Figure 5B), Ile-421 was involved in two salt bridges (Phe420 and His422) and a single hydrogen bond (His-X-His). Therefore, several factors, including constant salt bridge and hydrogen bond interactions, analogous secondary and tertiary structures, and the presence of an acidic group, might be reasons for the comparable enzymatic activity of I421A to CotA. Conversely, the K464A variant resulted in a substantial reduction in enzymatic activity. This phenomenon may be attributed to alterations in the hydrophobicity of the original structure (Figure S3). First, it was hypothesized that the interactions involving K464A and T466A were omitted, potentially impacting the configuration or dimensions of the substrate binding site. Then, the incorporation of an Ala residue was shown to induce alterations in internal passage folding [25], potentially leading to significant modifications in the structure surrounding the T2 and T3 copper site centers, as Ala is the simplest amino acid residue (Figure 5A). These alterations, in turn, may have resulted in a reduction in activity, which is possibly attributable to the shortening of molecular channels. In addition, the occurrence of hydrogen bond and salt bridge interactions was found to be diminished in instances where it was substituted with a nonpolar alanine residue [27] (Figure 5B). A similar disappearance of the hydrogen bond is observed at K464A. As illustrated in Figure 5B, the hydrogen bond initially located approximately 2.9 Å from the Asp279 and Glu298 in K464A has disappeared, clearly being observed to have moved away from the trinuclear copper cluster. This hindrance has the potential to affect the passage of water molecules toward the trinuclear copper cluster. In contrast, the amino acid Thr466 site enhances enzyme activity, which is presumably because of its interaction with Asp465. According to the findings of previous studies on CotA, Asp465, located in the exit of the water channel, has been identified as a key influential group in the neighborhood of the trinuclear center due to its steric hindrance [28]. The hypothesis suggests that substituting the amino acid residues around Asp465 with less hindered, conserved residues may allow a solvent molecule to interact with the copper ion. The reduction of the hydroxyl group has been identified as the rate-limiting step in the turnover of multi-copper oxidases [29].

Laccase expression, especially in fungi, is influenced by multiple factors, including the addition of copper ions [2,14]. However, the impact of substitutions in amino acid residues on the expression levels of bacterial laccases has received little attention. In this study, the SDS-PAGE results indicated that single-site substitutions in amino acid residues did not result in alterations in expression levels or affect cell biomass. However, with possible changes in folding or structural stability within the protein, the activity of enzymes may have changed due to their binding capacity with copper ions [30]. Additionally, purification of the target proteins revealed varying levels of soluble expression in response to different copper ion concentrations, as evidenced by Western blot analysis. It is hypothesized that lower concentrations of copper ions in CotA, I421A, and T466 expressions suffice for optimal protein folding and expression, resulting in a subsequent decrease in their overall expression. This is consistent with the research findings of Palm-Espling [31]. Cu^2+^ can specifically bind to unfolded and partially folded structures in vitro. It can both stabilize and disrupt the folded state of proteins. On the other hand, while copper ions have been observed to stimulate laccase synthesis, they concurrently exert an inhibitory effect on the proliferation of the strain [32,33]. It has been demonstrated that an increase in the concentration of copper ions results in a corresponding increase in the inhibitory effect. An excess of copper ions has been demonstrated to induce deleterious effects in biological systems [34]. The aforementioned phenomenon can be attributed to the prolongation of induction time, which results in the saturation of free Cu^2+^ required during laccase formation. This saturation leads to the gradual accumulation of residual Cu^2+^ within the strain, eventually culminating in its death and rupture. Conversely, it has been demonstrated that only copper ion concentrations greater than 1 mM can fulfill the expression requirements of K464A, as illustrated in Figure 2. Within the established range of grams (0.1 g), CotA and its various mutants exhibited a heightened requirement for elevated copper ion concentrations to achieve augmented levels of expression. CotA exhibited the most pronounced expression level, which is a consequence of evolutionary optimization over an extended period. In contrast, K464A exhibited a comparable expression capacity, indicating that K466 exerts minimal influence on the triple-cluster copper structure. Furthermore, the soluble expression levels of I421A and T466A exhibited a notable decrease. This reduction may be attributed to the increased proximity of these sites to the copper three-cluster anchor, which is a highly conserved region of the structure [35]. After replacing Ile421 with Ala, the side chain becomes shorter and the initial end of the central channel becomes wider, resulting in changes in the structure near the T1 copper position, which may affect enzyme folding efficiency. Thr466 is farther from the T1 copper than Ile421, so effective enzyme folding may be lower than that of the mutant I421A, leading to a lower laccase expression level. When Lys464 is replaced with Ala, the hydrophobic interaction force formed between the protein and water molecules is conducive to protein folding. Moreover, the Lys464 site is near the water molecule channel and is far from the T1 copper, so its influence on enzyme folding efficiency may be lower; thus, the expression level of the mutant K464A is higher. These minor structural alterations may have precipitated a modification in the microenvironment surrounding the copper ion landing site, thereby capturing copper ion influence during the folding process. Therefore, the enhanced functional expression may be attributable to the reduced size and minimal steric hindrance of the Ala side chain. This allows for the adoption of a broad spectrum of conformations [36,37]. We speculate that the mutation enhances the copper ion binding ability because the amino acid residues near the conserved amino acid residues around the copper ion have a shorter structure, which may increase the folding efficiency of the Cu^2+^ binding site, thereby helping to improve the binding ability of Cu^2+^.

The specific enzyme activity of I421A is similar to that of the wild-type CotA, and it is 1.06 times that of CotA. The amino group of the isoleucine side chain forms two hydrogen bonds with the adjacent carboxyl group of Ile421, as demonstrated by the structural analysis of the mutant I421A (Figure 5B). This observation persisted after the substitution of the amino acid with Ala. On the other hand, considering the Lys464 and Thr466 at the distal end of the water molecule channel, which are distant from the substrate binding site, it is hypothesized that they primarily influence the binding of water molecules to the three copper clusters rather than the binding of the T1 substrate. Consequently, the potential to affect the rate of water molecule formation is greater than the potential to influence affinity. Furthermore, K464A has been observed to alter the spatial configuration of the conserved Asp465 site within the WT-CotA, potentially impacting its biological activity (Figure S4). This phenomenon may cause a slight change in Asp465’s conformation with the three clusters of copper ligands, ranging from His422 to His424, compared with the WT-CotA. This finding underscores the critical role that the ligand structure surrounding the three copper clusters plays in determining the conversion number. As demonstrated in previous studies, the catalytic efficiency of the mutant T415G/T418I was found to be significantly reduced compared to that of the WT-CotA [38]. However, the T466A mutation exhibited varying effects on enzyme activity, which may be attributed to different amino acid residues interacting within the context of water molecule binding. The site Thr464, located at the edge of the exit channel, may demonstrate enhanced HO^−^ affinity for the channel due to modifications in hydrophobicity and polarity. Alternatively, this site may interact with another variable amino acid, Ser427, thereby increasing HO^−^ entry during catalytic turnover. This phenomenon bears a resemblance to previous studies [39]. Consequently, alterations in amino acid residues near the water channel can significantly impact enzyme activity [40].

The results demonstrate that all mutants except K464A exhibit an optimal temperature comparable to that of the WT-CotA. This finding indicates that changes in the internal residues of the channel have a minimal impact on the temperature requirements, as depicted in Figure S4. Furthermore, Lys464, located near the exit of the water molecule channel, may potentially aid in increasing the compactness of the protein surface, thus enhancing its hydrophobic properties, particularly in instances where hydrophobic amino acids, such as Ala, are substituted [39]. Furthermore, Ala has been shown to enhance hydrophobic interactions with surrounding water molecules [41]. The hydrophobic interactions of amino acid residues play a significant role in determining that the enzymes’ thermal stability of Ala can better fill the cavities in the protein core and enhance the hydrophobic stacking effect. A more compact and cavity-free hydrophobic core can more effectively resist thermal motion under high temperatures. In addition, Lys464, which possesses a positive charge, has the potential to engage in cation–pi interactions with other surrounding aromatic amino acids, such as Phe, Trp, and Tyr [42]. This interaction exerts a substantial influence on the stability of bacterial laccase. The optimal pH and the stability of the mutant enzyme and the WT-CotA are similar, indicating that the substitution of internal residues (Ile, Lys, Thr) does not significantly affect the optimal pH of the enzyme. At extremely high pH levels, excessive net positive or negative charges may cause intermolecular repulsion or attraction, leading to aggregation and precipitation. By mutating the charged residues on the surface to neutral residues (such as Ala), the extreme charge density on the surface can be reduced, allowing the enzyme to maintain solubility and monomer stability over a wider pH range [43,44].

CotA is regarded as a potentially effective bacterial laccase in the decolorization of dyes, which is a property attributable to its remarkable stability and tolerance [12,45,46]. These enzymatic properties fulfill disparate functions during the process of dye decolorization, and when confronted with distinct types of dyes, they exhibit varying oxidation capabilities. I421A and T466A achieved excellent decolorization of RB 19. In this study, Ile421 was found to be close to His422, which was identified as one of the axial ligands of the three copper clusters [47]. As demonstrated in previous studies, mutations in the active site have been shown to enhance the catalytic efficiency of the enzyme by facilitating electron transfer [48]. Therefore, the rate of electron transfer within structural molecules emerges as a notable factor affecting the dye conversion rate. In this case, Ile421 may serve as a key rate-limiting amino acid. Conversely, these sites are more likely to enhance the passage of water molecules and electron transfer, thereby increasing the dye decolorization effect. These phenomena may indicate that in addition to the dye’s affinity being relatively significant in the dye degradation mechanism, the rate of water molecule generation is also crucial, which could explain why the T466A mutant also has greater potential for dye decolorization [49]. A substantial body of prior research has demonstrated that by focusing on particular mutation sites, the decolorization capacity in the presence of dyes can be augmented [50,51].

## 5. Conclusions

In recent years, the CotA laccase from *Bacillus* sp., particularly in its role as a model system for studying the structure–function relationship of copper-containing proteins, has gained significant attention due to its unique properties and potential applications. For instance, research has shown that the CotA laccase can be effectively expressed in *E. coli* with certain secretion systems yielding high extracellular activity rates [52]. Furthermore, the enzyme’s stability and activity under various conditions, such as pH and temperature, have been characterized, revealing its robustness and potential for industrial use, such as in the degradation of mycotoxins. This heightened interest has illuminated its significant potential for application in biotechnology, particularly in the domains of delignification and dye degradation. Despite its status as an essential component of laccase, using copper ions as an inducer has not been fully explored. In this study, we examined the impact of incorporating copper ions on the expression of CotA with a single amino acid replacement. This study can serve as a foundational reference for the industrial application of the CotA laccase and its mutants. Incorporating Cu^2+^ and integrating protein engineering have been demonstrated to further enhance industrial catalytic efficiency. The industry’s pursuit of enhanced industrial catalytic efficiency should not be limited to the catalytic site of laccase. It has been demonstrated that altering other laccase sites can also significantly improve industrial catalytic efficiency. Our research findings emphasized the crucial role of amino acid residues adjacent to the conserved region in determining enzyme activity and functional expression. One hypothesis suggests that the improved functional expression could be due to an increased ability to bind copper ions. The functional expression delineated herein facilitates the generation of new variants through mutagenesis studies. Concurrently, the substantial decolorization capacity exhibited by the CotA suggests potential for a biological function involving the regulation of critical sites. Subsequent studies will focus on the dye decolorization of the obtained mutants under a range of conditions as well as the analysis of multiple-site mutations and substitutions. For instance, studies were conducted on the amino acid residues surrounding the Ile421 region of more electron channels. These investigations aim to provide further insights into the dye decolorization ability, solubility, and stability of the mutants. A subsequent investigation will be conducted to further explore the dye decolorization ability of the CotA laccase and the influence of protein engineering on the CotA laccase.

## Figures and Tables

**Table 1:** Mutation and WT–CotA laccase protein enzyme activity ratio.

Laccase	Enzyme Activity (U/mL)	Protein Content (mg/mL)	Enzyme Activity (U/mg)
CotA	1735.9 ± 63.35	0.1	17,359.4 ± 633.5
I421A	1842.2 ± 32.67	0.1	18,421.6 ± 326.7
K464A	3970.0 ± 102.42	0.1	39,700.2 ± 1024.2
T466A	1041.4 ± 89.32	0.1	10,414.0 ± 893.2
C492A	10.3 ± 4.82	0.1	103.1 ± 48.2

## Data Availability

The original contributions presented in this study are included in the article/Supplementary Material. Further inquiries can be directed to the corresponding authors.

## Supplementary Materials

The following supporting information can be downloaded at https://www.mdpi.com/article/10.3390/biology14101335/s1, Table S1: Construction of CotA mutants. Figure S1: SDS-PAGE analysis of the variants of laccase enzyme. Figure S2: The original image of Western Blot in Figure 2. Figure S3: The effects of temperature and pH on the activity and stability of wild-type CotA and mutant purified using ABTS as substrate. Figure S4: The K464A and T466A mutations are located at the tail of the water molecular channel in CotA, where O_2_ is converted to H_2_O. Figure S5: Analysis of the structural changes before and after K464A and T466A mutations.
