# Supplementary material for: Engineering the Bacterial Laccase CotA for Functional Expression and Dye Decolorization Through Site-Directed Mutagenesis
Source: Biology (Basel). 2025 Sep 28;14(10):1335. doi: 10.3390/biology14101335 (PMC12561958; doi:10.3390/biology14101335)
Supplement: Supplementary file 1 [file biology-14-01335-s001.zip › biology-3869194-supplementary.pdf]

Supplementary Material

Supplemental Table S1: Construction of CotA mutants, The bolded parts indicate the mutation positions.

Residue and mutation	Primers
I421A	F 5′-CGCGGAACACATCCG**GCA**CACCTGCATCTAGTC-3′<br>R 5′-GCGCCTTGTGTAGGC**CGT**GTGGACGTAGATCAG-3′
T466A	F 5′-AAGGGCTGGAAAGAC**GCA**ATTCAAGCGCATGCA-3′<br>R 5′-TTCCCGACCTTTCTG**CGT**TAAGTTCGCGTACGT-3′
K464A	F 5′-AGTGAAAAGGGCTGG**GCA**GACACCATTCAAGCG-3′<br>R 5′-TCACTTTTCCCGACC**CGT**CTGTGGTAAGTTCGC-3′
C492A	F 5′-CGATACGTATGGCAT**GCA**CATATTCTAGAGCAT-3′<br>R 5′-GCTATGCATACCGTA**CGT**GTATAAGATCTCGTA-3′

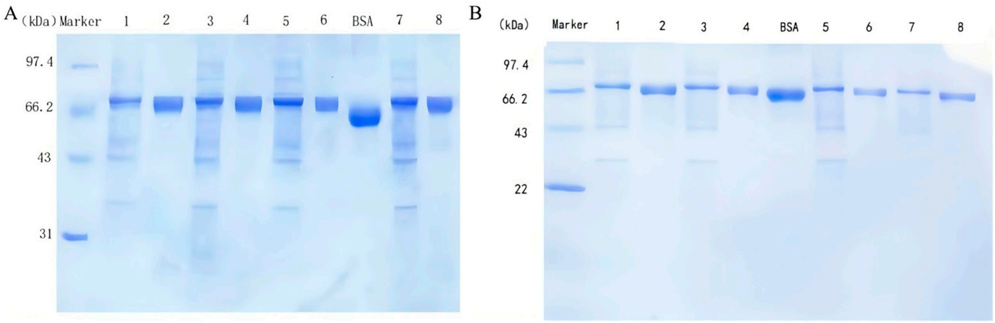

Figure S1: SDS-PAGE analysis of the variants of laccase enzyme (A) Same as the weight of the organism (B) Same volume of the bacterial solution; Protein samples were loaded in each lane as follows:(1) crude CotA; (2) purified CotA ; (3) crude I421A; (4) purified I421A; (5) crude T466A; (6) purified T466A; (7) crude K464A; (8) purified K464A.

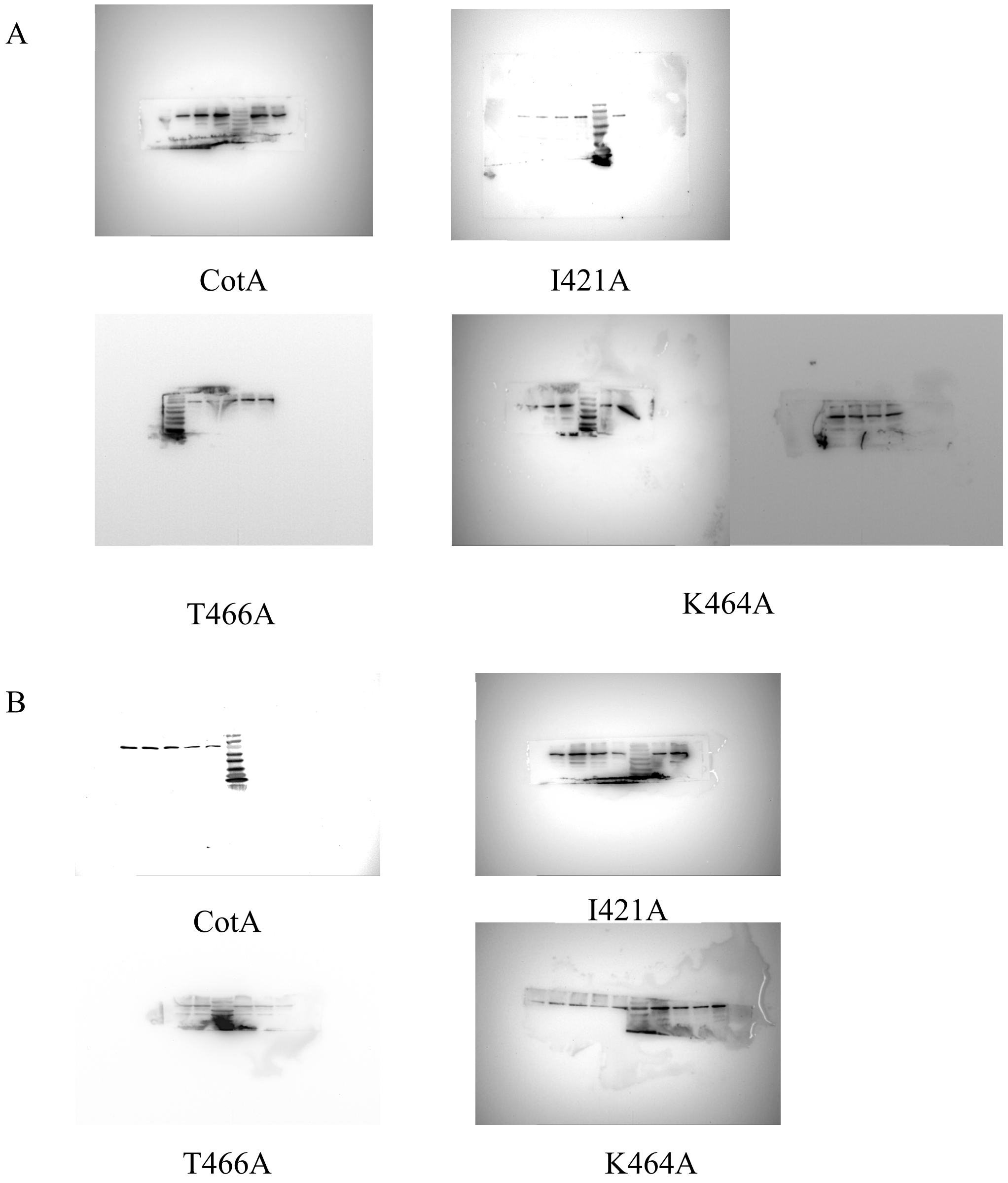

Figure S2: The original image of Western Blot in Figure 2. (A) Strain of the same weight. (B) Same volume bacterial strain.

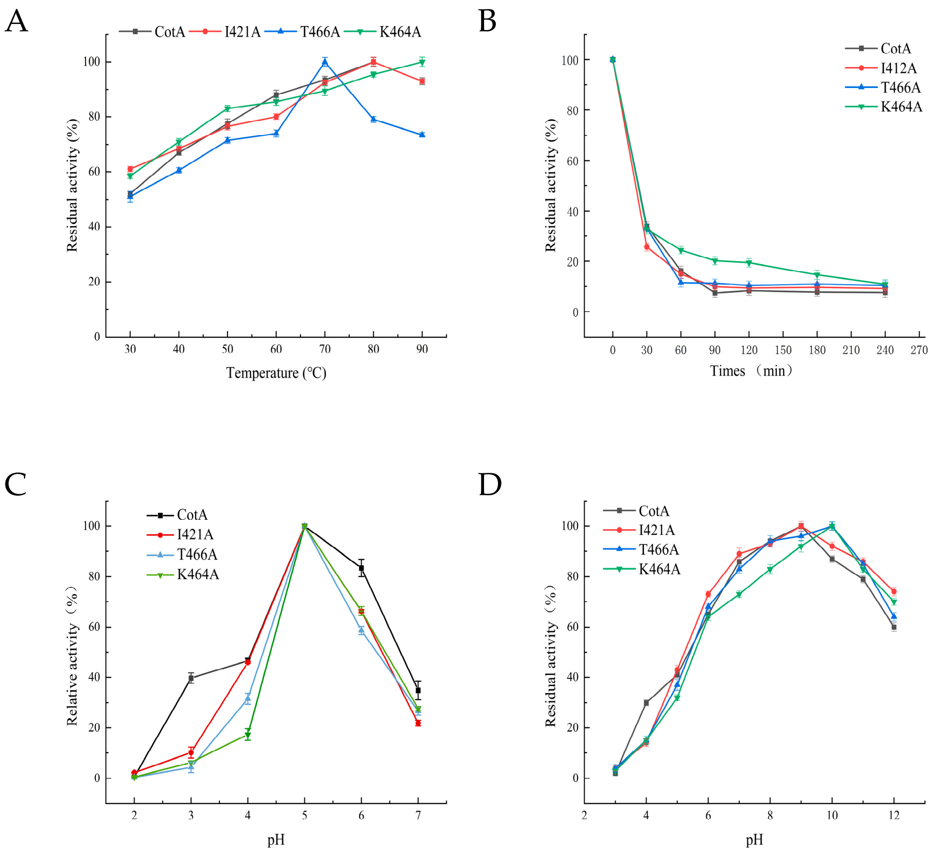

Figure S3: The effects of temperature and pH on the activity and stability of wild-type CotA and mutant purified using ABTS as substrate. (A) Measure laccase activity at different temperatures (30-90°C). (B) The mutant CotA laccase was determined after three high-temperature incubations at 60°C (C) Enzyme activity was measured using citric acid/phosphate buffer (100 mM) at pH 2.0-7.0. (D) After pre-incubation at different pH values (3.0-12.0) for 120 minutes, the activity was maintained at the optimal catalytic pH.

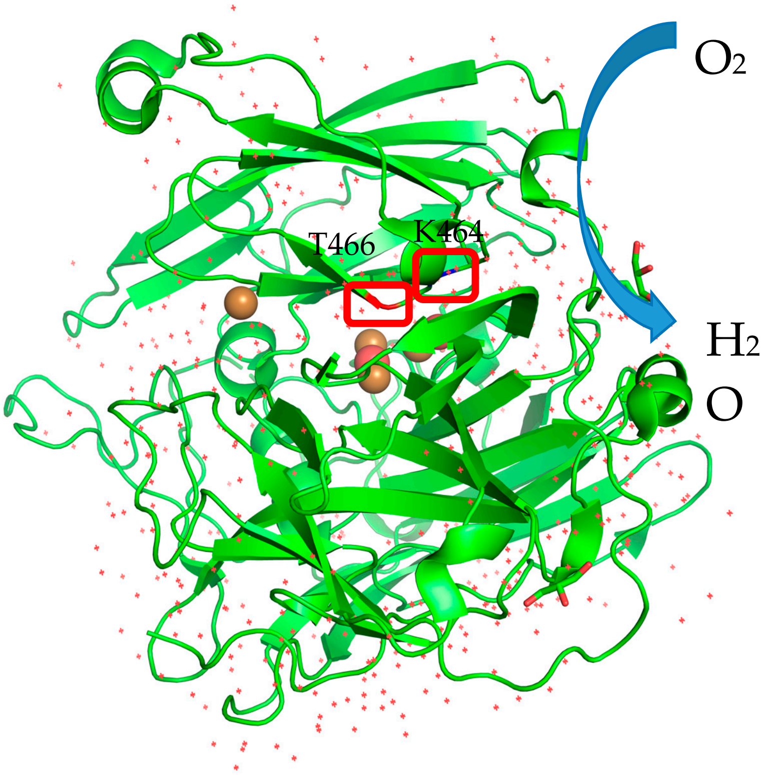

Figure S4: The K464A and T466A mutations are located at the tail of the water molecular channel in CotA, where O_2 is converted to H_2O. The T466A is highlighted in red. The K464A is marked in blue. The red dots represent water molecules.

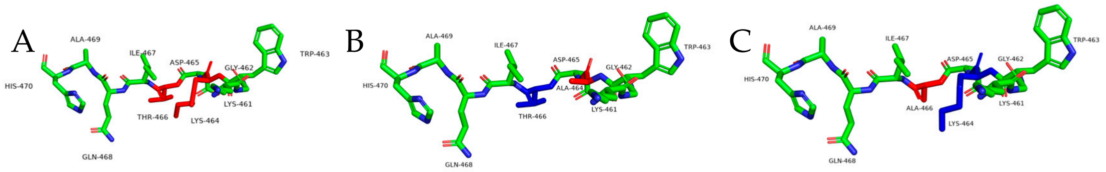

Figure S5: Analysis of the structural changes before and after K464A and T466A mutations (A) Both T464A and T466A are indicated in red (B) K464A is indicated in red and T466A in blue (C) K464A is indicated in red and T466A in blue
